# Supplementary material for: Scoping review of qualitative studies investigating reproductive health knowledge, attitudes, and practices among men and women across Rwanda
Source: PLoS One. 2023 Mar 31;18(3):e0283833. doi: 10.1371/journal.pone.0283833 (PMC10065244; doi:10.1371/journal.pone.0283833)
Supplement: S1 Appendix — (DOCX) [file pone.0283833.s001.docx]

**Appendix 1**

Search conducted by Gurpreet K. Rana, MLIS

Taubman Health Sciences Library

University of Michigan

Search strategies implemented on 3/3/2022

| **Database** | **Number of references** | **Deduplication done in EndNote** |
| --- | --- | --- |
| Medline | 165 |  |
| Embase | 178 |  |
| Scopus | 134 |  |
| CINAHL | 123 |  |
| Web of Science | 167 |  |
| Global Health | 115 |  |
| PsycINFO | 47 |  |
| Women’s Studies International | 19 |  |
| **Totals** | **Total number of citations before deduplication:**  **948** | **Final after deduplication (using EndNote dedup feature): 379** |

Total full-text reviewed: 82

Total excluded after full text review: 46

| Wrong population | 16 |
| --- | --- |
| Wrong study design | 4 |
| Wrong publication type | 14 |
| Wrong focus area | 9 |
| Wrong location | 1 |
| Does not report Rwanda-specific data separately | 2 |
| Total excluded after full text review | 46 |

Total included: 36, 3 found through hand-searching reference lists

Included domains:

| FP | 12 |
| --- | --- |
| SRHR | 23 |
| CAC | 1 |
| Total included | 36 |
