# Supplementary material for: Scoping review of qualitative studies investigating reproductive health knowledge, attitudes, and practices among men and women across Rwanda
Source: PLoS One. 2023 Mar 31;18(3):e0283833. doi: 10.1371/journal.pone.0283833 (PMC10065244; doi:10.1371/journal.pone.0283833)
Supplement: Attachment — Submitted filename: Response to Reviewers.docx [file pone.0283833.s003.docx]

23 February 2023

PLOS ONE Editorial Board

**Title: "Scoping review of qualitative studies investigating reproductive health knowledge, attitudes, and practices among men and women across Rwanda” (PONE-D-22-34373)**

Dear Md. Ashfikur Rahman:

We are very pleased to have the opportunity to revise our original manuscript for publication in *PLOS ONE*. We addressed each of the reviewer’s comments and made the necessary revisions and modifications. Responses are provided below.

**Journal Requirements:**

When submitting your revision, we need you to address these additional requirements.

**Requirement #1:** Please ensure that your manuscript meets PLOS ONE's style requirements, including those for file naming. The PLOS ONE style templates can be found at 
<https://journals.plos.org/plosone/s/file?id=wjVg/PLOSOne_formatting_sample_main_body.pdf> and 
<https://journals.plos.org/plosone/s/file?id=ba62/PLOSOne_formatting_sample_title_authors_affiliations.pdf>.

**Response #1:** Thank you, we ensured that our manuscript meets PLOS ONE's style requirements and updated the file names.

**Requirement #2:** We noticed you have some minor occurrence of overlapping text with the following previous publication(s), which needs to be addressed:

-https://www.unfpa.org/sexual-reproductive-health?page=3&type_1=All
-https://fp2030.org/sites/default/files/Rwanda%20FP-ASRH_Strategic%20Plan%202018%20-2014%20final.pdf

In your revision ensure you cite all your sources (including your own works), and quote or rephrase any duplicated text outside the methods section. Further consideration is dependent on these concerns being addressed.

**Response #2:** Thank you, in our revision we revised the overlapping text for UNFPA and FP2030 citations. We also ensured that we cited all our sources outside the methods section.

**Requirement #3:** Please include captions for your Supporting Information files at the end of your manuscript, and update any in-text citations to match accordingly. Please see our Supporting Information guidelines for more information: <http://journals.plos.org/plosone/s/supporting-information>.

**Response #3:** We updated the text to include captions at the end of the manuscript for the Supporting Information files.

**Reviewer comments:**

**Reviewer #1**

**Comment #1:** In ‘Introduction’ a clear explanation is needed about the background of the study and the importance of this study (Line no.124).

**Response #1:** Thank you for your comment. To improve the manuscript, a statement was added at the beginning of the introduction to summarize the need and importance of the study, which was also integrated throughout the introduction.

**Comment #2:** The heading of methods should be materials and methods according the guideline of PLOS ONE Journal (Line no. 157).

**Response #2:** Thank you for sharing this observation. We updated the heading of this section to adhere to submission guidelines.

**Comment #3:** In the ‘method’ section, the authors have mentioned that the search was limited to articles published from 2002 to 2022 (Line no. 175-176). So the authors should provide the logic of limiting the search within this timeframe.

**Response #3:** We appreciate this suggestion for improvement and revised the text providing the logic of limiting the search within this timeframe.

**Comment #4:** Under the sub-heading of ‘Screening Process’, the authors mentioned that the title and abstract screening was performed by two independent researchers (Line no. 194-195). So they have to specify who actually performed the title and abstract screening process. Similarly, the authors have to clearly specify who actually reviewed the full-text articles instead of using and/or vague term like (JMB, EA, OT, FGD, RG, GK, LI, TE, 197 and/or YS) (Line no. 196-197).

**Response #4:** We updated the text to mention who performed the title and abstract screening process. We also removed the vague term and/or to clarify that all authors listed in brackets actually reviewed the articles.

**Comment #5:** The authors are suggested to include the ‘Study quality assessment/Quality assessment of the included literature’ point under ‘materials and methods’ section (Line no. 157). They can follow the critical appraisal skill program (CASP) checklists (CASP, 2018) to assess the quality of the qualitative studies.

**Response #5:** We updated the text to incorporate this suggestion and indicated that we followed the CASP qualitative study checklist.

**Comment #6:** The ‘result’ section has to revise and extend in line with the specific objectives. It would be better to add relevant quotations (if possible) from included studies under different themes (Line no. 204).

**Response #6:** We thank the reviewer for sharing this suggestion for improvement. We revised the ‘result’ section by adding relevant quotations throughout from included studies under different themes.

**Comment #7:** The ‘Discussion’ section needs major revision. Here, the authors can compare and contrast their study findings with previous studies more extensively (Line no. 312).

**Response #7:** We expanded the discussion to compare our study findings with previous studies more extensively.

**Comment #8:** The authors have to revise the conclusion and have to specify the results-specific policy recommendations (Line no. 334).

**Response #8:** Thank you, we revised the conclusion and specified the results-specific policy changes recommended.

**Comment #9:** Overall, the writing of the manuscript has to improve following Standard English and ‘PLOS ONE’ Journal’s guidelines.

**Response #9:** Thank you, we revised the text throughout to improve English language usage and ‘PLOS ONE’ submission guidelines.

Sincerely,

The Authors
